# Stakeholders’ perspectives on facilitators of and barriers to the utilisation of and access to maternal health services in Eritrea: a qualitative study

**DOI:** 10.1186/s12884-018-1665-9

**Published:** 2018-01-19

**Authors:** Chol Chol, Cynthia Hunter, Berhane Debru, Berhana Haile, Joel Negin, Robert G. Cumming

**Affiliations:** 1 School of Public Health, The University of Sydney, Sydney, Australia; 2 Senior Lecturer, School of Public Health, The University of Sydney, Sydney, Australia; 3 Director General of Research and Human Resource Development, Ministry of Health, Asmara, Eritrea; 4 Director of Family and Community Health, Ministry of Health, Asmara, Eritrea; 5 School of Public Health, The University of Sydney, Sydney, Australia; 6 School of Public Health, The University of Sydney, Sydney, Australia

**Keywords:** Health system, Health system building blocks, Maternal health services, Gender equality, Female empowerment, Armed conflict, War

## Abstract

**Background:**

Wars affect maternal health services by destroying health systems. Eritrea experienced two wars with neighbouring Ethiopia. Despite this, the maternal mortality ratio (MMR) in Eritrea fell by 69% from 1590 per 100,000 live births in 1990 to 501 in 2015. This study aimed to examine facilitators of and barriers to the utilisation of and access to maternal health services in Eritrea.

**Methods:**

Using in-depth interviews and field observations for data collection, this qualitative study was conducted in five healthcare facilities in Asmara, the capital of Eritrea, in February and March 2016. The participants were: women (*n* = 40), husbands (*n* = 5), healthcare providers (*n* = 10), and decision makers (*n* = 5).

**Results:**

There were two perceived facilitators of utilisation of and access to maternal health services: health education (related to the WHO health service delivery building blocks) and improvement in gender equality driven by the role played by Eritrean women as combatants during the War of Independence (1961–1991). The only perceived barrier was poor quality of care due to lack of ultrasound machines, short clinic opening hours, and shortage of healthcare workers (related to the WHO health workforce building block).

**Conclusion:**

This study assessed women and their husbands/partners’ perceptions and the possible effects of contemporary Eritrean culture and the history of war on the utilisation of and access to maternal health services in the country. As well, we examined healthcare providers’ and decision makers’ perspectives. The two key facilitators of women’s utilisation of and access to maternal health services were health education and women’s empowerment driven by their role as combatants during the War of Independence. One main barrier was poor quality of care due to lack of ultrasound machines, short clinic opening hours, and a shortage of healthcare workers. As only a limited number of qualitative studies have been published about maternal health services in war-affected sub-Saharan African countries, our findings regarding health education and women’s empowerment could be considered in other war-affected countries similar to Eritrea. Nevertheless, further research is needed to investigate our findings – particularly regarding female empowerment driven by women’s role in combat in relation to their maternal health.

## Background

Wars destroy the health systems responsible for promoting, restoring and maintaining delivery of healthcare and provision of maternal health services [1–5]. Recent studies have shown that between 1989 and 2011, Africa and particularly sub-Saharan Africa (SSA) experienced 46 armed conflicts, more conflicts than in any other region in the world [6, 7]. As well, in 2015 SSA had the highest maternal mortality ratio (MMR) world-wide [8].

Eritrea is one of the SSA countries that have experienced a long history of war, and it also has a high MMR [8–11]. Eritrea experienced two wars with neighbouring Ethiopia: during 1961–1991 (the War of Independence) and 1998–2000 [9, 10]. Women formed 33% of the combatants during the War of Independence [12]. After independence, Eritrea had a very high MMR of 1590 which was reduced to 501 by 2015: a 69% reduction [8]. This MMR reduction could be a result of numerous policies such as the Post-Independence Macro Economic Policy Framework which included a vision to improve access to healthcare facilities [13–15]. As a result of this policy, the number of hospitals increased from 16 to 28, health centres from four to 56, and the number of health stations increased from 106 to 188 during 1990–2013 [13, 14].

Eritrea attempted to solve the shortage of healthcare workers by increasing the number of nurses and by encouraging task shifting. Between 2002 and 2012, the number of nurses increased from 846 to 1253 and associate nurses (diploma and certificate) from 1488 to 2537 [13]. Task shifting to solve shortage of healthcare workers also involved combining midwifery and nursing training as well as employing foreign doctors from countries such as Cuba [13, 16, 17].

Challenges still exist in Eritrea’s health system. Only a small percentage of women deliver in health facilities [14]. Despite 57% of Eritrean women having the WHO recommended four antenatal care (ANC) visits by 2015 (compared to SSA average of 48%), only 34% of women delivered in health facilities in 2010 compared to 17% in 1995 – below the SSA average of 48% [18–20]. Moreover, there is a large gap in facility based deliveries between urban and rural areas. In 2010, 73% of women living in urban areas delivered in a health facility compared to only 27% of rural women [18–20].

This study aimed to examine stakeholders’ perspectives on facilitators of and barriers to the utilisation of and access to maternal health services in Eritrea, focusing particularly on women’s perspectives. The objectives were: (1) to assess women and their husbands/partners’ perceptions about the facilitators of and barriers to the utilisation of and access to maternal health services; (2) to examine the possible effect of Eritrean culture on utilisation of and access to maternal health services; (3) to explore how the two wars (1961–1991, 1998–2000) have influenced women’s status and affected their utilisation of and access to maternal health services; and (4) to investigate perspectives of healthcare providers and decision makers regarding maternal health services.

## Methods

### Setting and sampling

This study was conducted over a three-week period during February and March 2016 in Asmara, the capital of Eritrea, in four ANC clinics and one maternity ward. Asmara was chosen because its health facilities receive the largest number of outpatient visits in the country [13]. The four selected ANC clinics were located in Godaief Community Hospital, Edget Health Centre, Freesalam Clinic and Akreya Clinic. The maternity ward was located in Orotta Teaching Hospital. These health facilities were purposively selected based on their demographic characteristics. Edget Health Centre was chosen because it is located in a predominantly Muslim area, and thus could aid investigation into whether religion [21] and polygamy [22] hinder women’s utilisation of and access to maternal health services. Freesalam Clinic was selected because it is in an affluent area and Akreya Clinic was chosen because it is in a socially disadvantaged area. Godaief Community Hospital was selected because it is a zobal (regional) hospital and receives patients from health stations and health centres in rural areas. Finally, Orotta Teaching Hospital maternity ward receives high risk women and complicated maternal cases.

The health system in Eritrea is divided into three levels [23]. Primary level healthcare is provided by community hospitals, health centres, and health stations. Community hospitals provide preventive and curative services and receive referrals from health centres and health stations in rural areas. Secondary level healthcare is provided by zobal (regional) referral hospitals which provide inpatient and outpatient services for the whole zoba and receive referrals from primary level health facilities. This level is also responsible for community mobilisation. Finally, tertiary level healthcare is provided by the national referral hospitals located in the capital Asmara: Orotta Teaching Hospital, Halibet Hospital, Berhan Eye Hospital, and St Mary’s Psychiatric Hospital. Until 2014 there was private healthcare in the country [24]. However, by 2016, when this research was conducted, the government had abolished privately owned clinics and replaced them with its own out-of-pocket private health services within the public healthcare system.

### Participants selection

The majority of our participants were women aged between 15 and 49 years who were pregnant at the time of the research, or had given birth between February and March 2016 (Tables 1 and 2). “Women or female participant” in the quotes hereafter, refers only to women who used maternal health services in our sample excluding female decision makers and healthcare providers. The second group of our participants were husbands/partners. They were included because their role has been found elsewhere in SSA to have an impact on women’s utilisation of and access to maternal health services [25]. Finally, we interviewed healthcare providers and decision makers as key informants to highlight any health system challenges. They were recruited using purposive sampling based on their expertise, involvement in and knowledge of maternal health services [26].

**Table 1:** Characteristics of women participants (*n* = 40)

Characteristic		Number
Ethnicity	Tigrigna	39
	Saho	1
Age (years)	15–16	1
	17–24	11
	25–34	25
	35–49	3
Education	Primary school (grades 0–7)	9
	Middle school (grades 8–10)	15
	Secondary school (grades 11–14)	14
Occupation	Unemployed	35
	Employed	5
Religion	Orthodox Christian	23
	Other Christian	5
	Muslim	8
Marital status	Never married	1
	1st wife	36
	2nd wife	2
	3rd wife	1
Parity (the number of times a female is or has been pregnant (gravidity) and carried the pregnancies to a viable gestational age)	0	13
	1	1
	2–3	9
	4–7	11
Number of abortions		7

**Table 2:** Characteristics of husbands, healthcare providers and decision makers

Location		Husbands (*n* = 5)	Healthcare providers (*n* = 10)		Decision makers(*n* = 5)
			Nurses/midwives (*n* = 9)	Doctors^a^ (*n* = 1)	
Healthcare facilities	Freesalam Clinic	1	2		
	Akreya Clinic	2			
	Edget Health Centre	2	2		
	Godaief C. Hospital		4		
	Orotta Teaching Hospital		1	1	
Ministry of Health	Family planning				1
	Human resources				1
	Research				1
	Data management				1
	FGM^b^ programme				1

^a^Head of gynaecology department ^b^Female genital mutilation

All women and their husbands/partners were approached for recruitment in the general waiting areas and courtyards (that is, away from any of the healthcare providers) to avoid coercion. The lead researcher (CSC), with the help of research assistants, selected the women invited to participate within this cohort. Interviews with the women usually took place in private rooms or quiet courtyards within the health facilities. Although all 40 women were given the option to be interviewed after their consultations, 12 women chose to be interviewed in waiting rooms. These were large rooms where healthcare providers conducted consultations with other women and also contained multiple desks and partitions. We conducted the interviews with the 12 women on benches positioned so that women’s privacy was maintained, being far away from healthcare providers so that they could not hear the interviews. Husbands/partners were recruited and interviewed in ANC clinics’ courtyards while their wives/partners were having their ANC consultations. Finally, healthcare providers and decision makers were selected as key informants by the lead researcher. Interviews with healthcare providers were conducted in the health facilities early in the morning or at the end of the day to avoid busy consultation hours. Decision makers were interviewed by the lead researcher in their offices at the Ministry of Health at the national headquarters.

### Conceptual framework

**Fig. 1:**
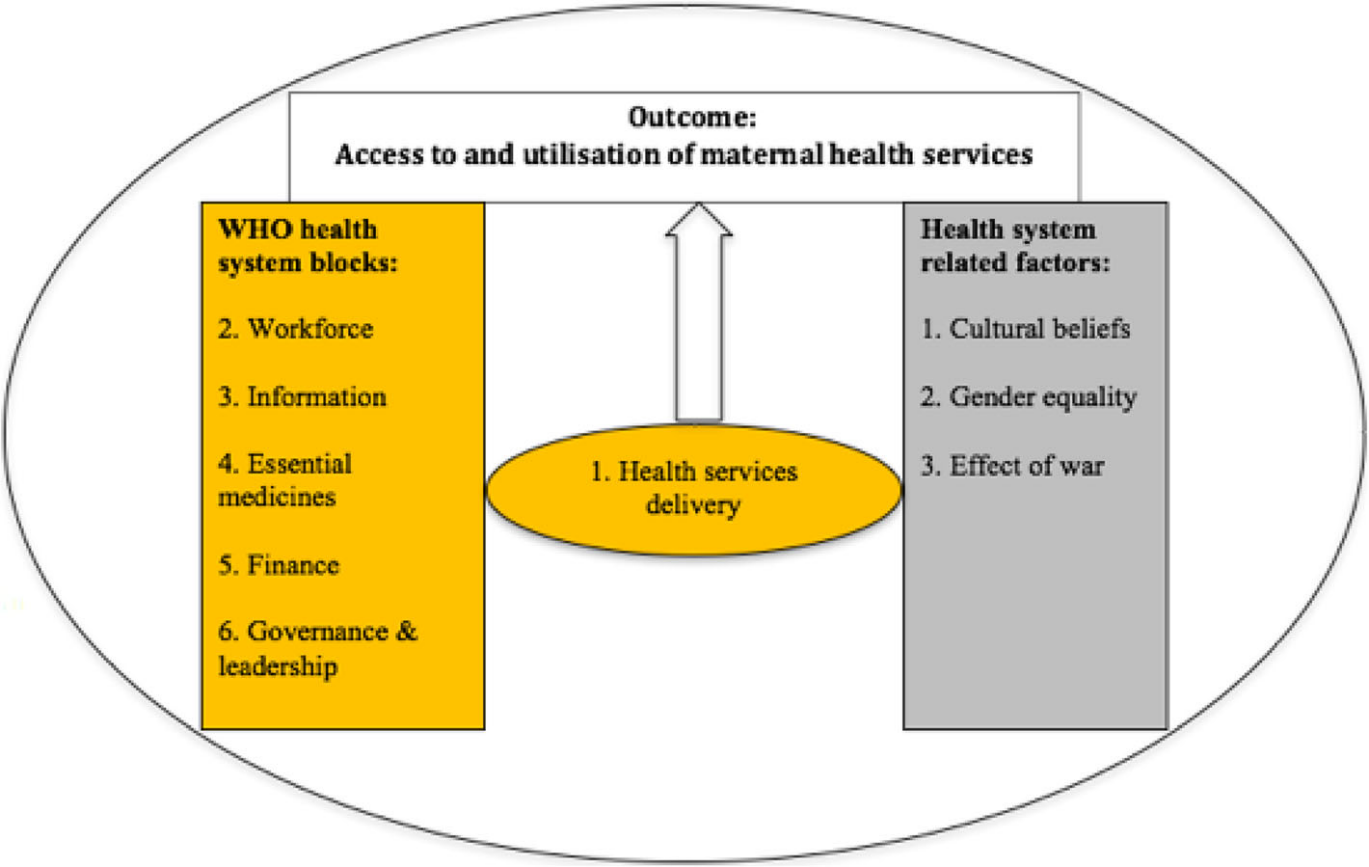
Conceptual framework: Factors affecting women’s utilisation of and access to maternal health services in Eritrea

There are many factors that have been found to affect women’s utilisation of and access to maternal health services. However, the focus of this study was factors associated with the WHO six health system building blocks, as well as an additional three factors [27–29], as shown in Fig. 1. The WHO six health system building blocks are broad indicators used to measure health system performance: (1) health services delivery; (2) health workforce; (3) health information system; (4) essential medicines; (5) health finance system; and (6) leadership and governance [27]. The six health system building blocks are also concerned with how stakeholders’ perspectives and satisfaction could be used to measure health services delivery [27].

Figure 1 shows our adapted framework based on the WHO framework with an additional three factors not incorporated in the WHO framework: cultural beliefs, gender equality, and war. Cultural beliefs and gender equality were added because they have been found to affect women’s health [28, 29]. War was added to the framework because it was one of our study objectives and has been found to negatively affect women’s utilisation of and access to maternal health services [30–32].

### Data collection

To generate knowledge about maternal health services in Eritrea, we collected data through in-depth interviews and field observations. For the in-depth interviews, identical open-ended questions were used for each group of participants (see supplementary material for our interview guidelines). The lead researcher conducted interviews with all healthcare providers and decision makers in English. The rest of the interviews were conducted with help from or by local research assistants – a male and two females in their seventh year of medical school who had had prior experience in conducting qualitative research. Research assistants who were fluent in both English and the main language spoken in the country (Tigrigna) were coached by the lead researcher about conducting qualitative research face to face. Research assistants then used the Tigrigna language to explain the selection criteria, confidentiality, disclosure of information and the aim of the study to participants before obtaining their consent.

All interviews were conducted one-on-one. As participants did not want the interviews to be recorded using a voice recorder, extensive note taking was used to record the interviews. Each interview lasted between 20 and 45 min, and stopped when no new ideas emerged. For the field observations, the lead researcher used extensive note taking that included documenting healthcare providers’ interactions with women such as the consultation process, how healthcare providers recorded patients’ information, and the general environment of the health facilities – such as their level of cleanliness.

### Data analysis

Data analysis proceeded in several stages. First, when possible, the transcripts were read multiple times by the lead researcher and discussed face-to-face with research assistants on the same day as the interviews. During these daily discussions between the lead researcher and research assistants, field observation notes were also used to cross examine and interpret our in-depth interviews [33]. The aim of this stage of the process was to better understand the cultural context of the quotes beyond the obvious meaning or direct translation. Second, the qualitative information that was collected was subsequently used to inductively generate theory from the raw data (using Microsoft Excel) about health system performance and maternal health services [34, 35].

During the second stage of analysis, themes were inductively generated in three steps. First, a unique colour was assigned to words and/or phrases with similar meanings (codes) that were linked to the study objectives and prior knowledge of the literature review about Eritrea. Second, codes were assigned to each segment of the transcript in order to categorise the main concepts and link emerging data to research objectives while keeping the original phrases and context [34, 36–38]. Third, repeated codes were grouped into themes, examined for consistency, and discussed with co-author (CH). Finally, themes were grouped under the appropriate category and used for the final interpretation of the data.

### Ethical approval and consent to participate

Ethics approval was obtained from the Health Research and Ethics Committee, Ministry of Health, The State of Eritrea. The Ethics Committee approved either written or oral consent to be obtained from the participants including minors’ guardian. As our study included a 16 year old participant, her mother gave an oral consent for her participation and to use her quotes in this study. All participants gave oral consent for this study and were recorded by their initials only. No incentives or any form of financial compensation was given to any of the participants. This was explained to all participants during the recruitment process.

## Results

In this study, 60 in-depth interviews were conducted: women (*n* = 40); husbands/partners (*n* = 5); healthcare providers (*n* = 10) (nine females and a male); and decision makers (*n* = 5) (three females and two males). Characteristics of participants are shown in Tables 1 and 2. Table 3 shows perceived facilitators of and barriers to women’s utilisation of and access to maternal health services in Eritrea.

**Table 3:** Women’s perspectives regarding perceived facilitators of and barriers to maternal health services in Eritrea (*n* = 40)

Facilitators	Barriers
Health education:	Perception of quality of care:
Information related to:	A call for private healthcare:
• Importance of delivering in a health facility	Reasons:
• Danger signs (fever, vomiting, bleeding, discharge, persistent headache and/or dizziness)	• To have access to ultrasound scans (no ultrasound scans were performed on pregnant women who participated in this study in all antenatal care clinics we visited except in Orotta Teaching Hospital maternity ward)
• Breast check during shower for any lumps	
• Vaccination	
	• To decrease the workload on the public clinics and avoid crowdedness of the health facilities. Therefore, less waiting time.
	• To avoid short opening hours in the public clinics
Improvement in gender equality:	Previous hospital experience:
• Role of Eritrean women as combatants during the War of Independence	Reasons for dissatisfaction:
	• Staff shortage
• Improvement in men’s attitude towards maternity care	• Lack of sympathy
	• Poor treatment by healthcare providers
	• Not enough information in the postnatal period (6 weeks after childbirth)

Our study revealed two perceived facilitators of maternal health services in Eritrea: health education and improvement in gender equality. One major perceived barrier was women’s perception of poor quality of care (Table 3).

### Health education

We investigated the level of satisfaction expressed by women and their husbands/partners with the health information provided to them during their ANC visits. Thirty-four of the 40 women and all five husbands/partners we interviewed were satisfied. Women mentioned that they were encouraged to visit a health facility if they felt any of the danger signs during pregnancy: fever, vomiting, bleeding, discharge, persistent headache and/or dizziness (Table 3). The lead researcher observed that all five health facilities we visited had educational sessions each morning. These sessions were conducted by nursing cadres in the health facility courtyards, and provided an opportunity for nurses to discuss and answer health questions from pregnant women, husbands/partners and other family members. The lead researcher also observed that health education involved use of posters and signs raising awareness about maternal health issues in all healthcare facilities we visited.

*“It is helpful* [health education] *in a way that it encourages you to follow up your health situation”* **Female participant from Edget Health Centre**

*“Information about variety of tests, about how to support women physically and mentally and supporting them to come to the clinic has big role”* **Husband from Freesalam Clinic**

However, six women wanted more health-related information. A mother who had given birth 2 days prior at Orotta Teaching Hospital maternity ward, complained about lack of information given to her in the hospital. One of the six women also complained about the lack of reinforcement of the information each time they visited ANC clinics.

*“No information was given about childbirth”* **Female participant from Orotta Teaching Hospital maternity ward**

*“My experience could have been better if the information given to pregnant women was given several times”* **Female participant from Edget Health Centre**

Although all five husbands/partners were satisfied overall with the health information given to them in the courtyard sessions, some of them complained that they were not able to accompany their wives/partners during their consultations with nurses/midwives. This was confirmed by the lead researcher who observed that during all ANC visits, women were seen separately from their husband/partner. The only exception observed was during the first trimester, when women and their husbands/partners are consulted together and tested for HIV and other infectious diseases.

*“Better if I learn more here* [at the ANC clinic] *with my wife, but I learn a lot by reading books”* **Husband from Akreya Clinic**

We investigated how contemporary cultural beliefs affect women’s utilisation of and access to maternal health services in the country. Although the government health education strategy to combat negative cultural practices such as Female Genital Mutilation *(*FGM) involves use of mass media, some participants mentioned that mass media could be improved to enhance the distribution of health messages. Of the 40 women we interviewed, 38 women indicated that contemporary culture in Eritrea encourages them to access and utilise maternal health services. One example that seems to indicate a degree of cultural change was the account told to us by a 16 year old mother interviewed in Orotta Teaching Hospital maternity ward about her decision to access health services. She spoke of her hesitation to attend an ANC clinic because of her extramarital pregnancy at a young age. However, she eventually gave birth in a healthcare facility and was supported by both her mother and her boyfriend.

*“Our culture and families motivate us to come to the clinic and follow our pregnancy”* **Female participant from Akreya Clinic**

*“I was afraid to come to antenatal clinic because I was not married and became pregnant…my mam and my culture motivated me to access maternal health services”* **Female participant from Orotta Teaching Hospital maternity ward**

*“During the war, we tried to stop FGM even during the war by putting policies in place…. our position as women is much better than before the war. We have more rights”* **Decision maker (female) from Ministry of Health**

*“FGM is practised by all ethnic groups regardless of religion. It is punishable by law with jail time and fine of 10,000 nakfa* [Eritrean currency]*. It is higher among Muslims compared to Christians. The reasons among Muslims is for cleanliness and among Christians is to maintain virginity”* **FGM programme decision maker (male) from Ministry of Health**

*“…. mass media needs to increase the awareness and improve the knowledge of men about pregnancy… It is not well understood that men have to support women, because society lacks awareness but I believe that I need to support my wife”* **Husband from Edget Health Centre**

Even though the majority of the participants indicated that contemporary cultural beliefs in the country encourage women’s utilisation of and access to maternal health services, some disagreed. For example, one woman indicated that the cultural preference is for women to deliver at home but that she prefers to deliver in hospital.

*“I believe cultural beliefs prefer that women deliver at home but I personally prefer to deliver in a hospital and go to a clinic while I am pregnant”* **Female participant from Godaief Community Hospital**

Community mobilisation – that is, community-driven activities where communities make autonomous decisions to improve their own health [39, 40] – was mentioned by a decision maker as being one of the factors that helped influence women’s utilisation of and access to maternal health services. However, our study could not substantiate this claim since no community driven health activities were observed or mentioned by most of the participants.

*“Community mobilization and making information available to them* [women] *is one of the most effective health policy strategies that helps reduce maternal mortality here”* **Decision maker (female) from Ministry of Health**

### Improvement in gender equality

#### Role of Eritrean women during the war of independence (1961–1991)

Women were asked how the history of wars affected women’s status and their utilisation of and access to maternal health services. All women mentioned that war helped to improve gender equality in the country. This improvement was mainly driven by their role as combatants during the War of Independence. For example, a former combatant now working as a nurse/midwife was interviewed, and showed the lead researcher marks of five healed bullet wounds in her lower limbs. She spoke of how she suffers from a permanent hip injury from the war and about her fighting experience alongside men during the War of Independence.

*“I was a fighter and a field doctor at the same time. First, I was shot twice during the war and while recovering, I gave birth to my eldest son in a mobile clinic. Women used to hide their pregnancies so they could be in the field fighting with men. When they were about to give birth, they were provided with a place to give birth and stay with their babies for one year and a half but some women sometimes chose to leave early to fight. After I gave birth to my son I used to look after other women’s children while they were fighting. I left my son after a year with the Sewera brigade* [revolution brigade responsible for looking after other women’s children while they are in the field fighting]*. After I went back to the war, I was again shot three times and suffered a permanent hip injury… and my husband was killed during the war* [emotional]*…I still believe in helping women to give birth safely…I agree with the government for men to pay if they do not come with their wives for check-up. We are the same now because we fight together. I struggled in the war like them* [men]*”* **Nurse/midwife (female) from Godaief Community Hospital. A former freedom fighter who also worked as a field doctor (nurse/midwife) during the War of Independence.**

*“The role of Eritrean women during the war was an important factor for gender equality in Eritrea….”* **Decision maker (female) from Ministry of Health**

*“My mother fought as a freedom fighter to make it better for me now. My husband helps me with cooking now because he know*s *we are the same. No difference now between a girl and a boy”* **Female participant from Freesalam Clinic**

*“During the war mothers were not able to get access to health centres as there were no adequate hospitals, medical supplies and workforce…. I am the same with my man now. My mother and my father liberated the country fighting together. Because of this we are the same now with my husband* [putting both index fingers alongside each other meaning equal]*”* **Female participant from Edget Health Centre**

*“After the war, women are more aware* [of their rights] *and free to go to hospitals and get service. There are more health centres* [now] *and better service”* **Female participant from Edget Health Centre**

#### Role of men during pregnancy and child delivery

We asked the participants about men’s role during pregnancy and childbirth. Twenty-three women would like their husbands/partners to help with housework and 13 women said they would like their husbands/partners to support them by being there during childbirth. All five husbands/partners who were interviewed agreed that men should support their wives/partners.

The involvement of men in their wives/partners’ maternity care has been addressed by the government through community education programmes [41]. For example, men are required to accompany their wives/partners in the first trimester to check for infectious diseases. In addition, men are required not to divorce their wives/partners during pregnancy.

*“It has been made compulsory for fathers to attend in the 1st trimester with their wives to check for STDs* [sexually transmitted diseases] *and HIV. Men are obligated to stay with their wives during this period of pregnancy and not to divorce”* **Nurse/midwife (female) from Freesalam Clinic**

*“My husband helps me a lot with housework and heavy lifting, he motivates me and he is here now. He learns with me”* **Female participant from Akreya Clinic**

*“Men should help women during pregnancy and when giving birth. My boyfriend helped me a lot during my pregnancy and now after I gave birth”* **A young unmarried female participant from Orotta Teaching Hospital maternity ward**

*“During pregnancy men should support mothers morally and help them with housework. During delivery men should stand beside mothers and share their pain”* **Female participant from Akreya Clinic**

*“Continue to increase awareness that a man needs to come to the clinic with his wife”* **Husband from Edget Health Centre**

### Quality of care

#### Preference for private healthcare (unexpected finding)

Privately owned health clinics existed in Eritrea until 2014, but by the time this research was conducted in 2016 they had been abolished by the government. Instead, the government provided its own out-of-pocket private health services within the public healthcare system. Our initial interview guidelines did not include a question concerning private healthcare because we were not aware of the demand for this service. However, a question about private healthcare was added when the issue emerged during our first interview when the first woman we interviewed said she would like private healthcare due to the lack of ultrasound machines in the government ANC clinics.

We asked women if they would like to see private healthcare reinstated, the reason for their choice, and whether they could afford it. Of the 40 women we interviewed, 31 expressed dissatisfaction with some aspects of the government services and stated that they would like private clinics reinstated. Their dissatisfaction was due to three reasons: lack of ultrasound machines, short clinic opening hours, and a shortage of healthcare workers. A lack of ultrasound machines was observed by the lead researcher: none of the five ANC clinics we visited had an ultrasound machine. The lead researcher also observed that most of the ANC clinics did have short opening hours as well as having long wait times: they opened at 7 o’clock each morning and closed at midday, with the average waiting time of 40 min. Finally, shortage of healthcare workers was observed by the lead researcher as a particularly significant concern in one of the ANC clinics, which had only one nurse/midwife (former freedom fighter) attending to 34 pregnant women on the day the clinic was visited.

*“It would be nice if there is private clinics to have ultrasound* [scan] *and I could afford it”* **Female participant from Freesalam clinic**

*“It is really good* [to have private healthcare]*. I would have been able to get a better concept about the health of my child, like doing ultrasound* [scan]*”* **Female participant from Godaief Community Hospital**

*“It is good that the private clinic exists, because it is better with timing and you can access it at any time”* **Female participant from Akreya Clinic**

*“Increase the workforce as this nurse is lone and has very big workload”* **Female participant from Edget Health Centre**

Although 31 women wanted private health care, nine women disagreed. They were satisfied with the current public services and they believed that private healthcare would be a waste of resources that could otherwise be spent to improve the existing public health services. They also mentioned that they would not be able to afford to pay for private health care.

*“No need* [for private healthcare]*. Because it is waste of time and money as the health professionals are more helpful and supportive at this health centre”* **Female participant from Godaief Community Hospital**

*“No, I don’t think private clinics should be available. It would be better to just improve the services given in the* [current] *clinics and hospitals”* **Female participant from Edget Health Centre**

*“Private clinics are quite expensive and it is hard to afford financially. So, I do not prefer private clinics…”* **Female participant from Godaief Community Hospital**

#### Previous hospital experience

Women were asked to describe their previous hospital experiences and their treatment by healthcare providers during childbirth or any maternal complications. This question was answered by 23 of the 40 women we interviewed. Seventeen women were excluded – four chose not to comment and 13 women had no comments to make as it was their first pregnancy. Fifteen women had had a positive previous hospital experience and eight women had had a negative experience. The dissatisfaction of the latter group of women was due to shortage of healthcare workers, poor treatment by healthcare providers, and long waiting times.

*“It is important that I went to the health centre to follow my pregnancy and I am so happy to deliver in this hospital”* **Female participant from Akreya Clinic**

*“The last visit was good. If it had not been for them* [healthcare providers]*, I would not have delivered a healthy baby because my baby had an abnormal presentation and the staff managed to help”* **Female participant from Freesalam Clinic**

*“We need health professional help during pregnancy and labour. Women should get access to delivery at hospitals”* **Female participant from Godaief Community Hospital**

*“I had a caesarean section. I could have given birth naturally if I had received adequate support. There were not enough health professionals around in the evening”* **Female participant from Edget Health Centre**

“*My delivery was successful but the midwives were not following my labour properly”* **Female participant from Akreya Clinic**

*“It is better that the health service providers understand that we have children to take care of at home, so better to manage the time and accessibility”* **Female participant from Freesalam Clinic**

## Discussion

This study set out to explore stakeholders’ perspectives (particularly women’s) on facilitators of and barriers to the utilisation of and access to maternal health services in Eritrea. Two major perceived facilitators that emerged were: health education and improvement in gender equality based on the Eritrean women’s role forming 33% of the armed forces during the War of Independence. One perceived barrier was poor quality of care, mainly due to lack of ultrasound machines, short clinic opening hours, and shortage of healthcare workers.

Out study revealed two findings related to the WHO health service delivery and health workforce building blocks. Health education – as a component of health promotion – was one of the facilitators we found related to the health service delivery building block. The second finding related to the health workforce building block was shortage of healthcare workers as a barrier. It is interesting that the majority of women did not mention affordability as a barrier to the use of maternal health services when they called for private healthcare to be reinstated in the country after it was abolished by the government in 2014. As well, they did not mention task shifting to solve shortage of healthcare workers or possible barriers associated with family composition, their financial situation or distance from healthcare centres.

### Perceived facilitators of maternal health services

#### Health education

We assessed the perceptions held by women and their husbands/partners regarding the facilitators of and barriers to maternal health services in Eritrea (objective 1). Health education (a component of health promotion) was the only facilitator related to the WHO health service delivery building block [27, 42]. Health promotion forms an essential part of comprehensiveness which is one of eight key characteristics of the health service delivery building block [27]. It is defined as “the process of enabling people to increase control over their health and its determinants, and thereby improve their health.” [42].

Health education in Eritrea takes two forms: education provided through health facilities and education enabled through the use of mass media. Health education in health facilities mainly involves sessions in the morning targeting women and their families. These sessions were acknowledged by 34 of the 40 women we interviewed as being effective in encouraging them to access and utilise maternal health services. Furthermore, 34 women expressed satisfaction with the health information given to them at the health facilities which encouraged them to visit a health facility if they experienced the five danger signs. Health education could also be one of the reasons for the increase in the percentage of women attending an ANC clinic at least four times – an attendance pattern recommended by the WHO as an essential requirement for maternal mortality reduction [18]. During 1995–2015, the percentage of Eritrean women attending an ANC clinic four or more times increased from 41% to 57%, compared to SSA average of 48% [18, 19]. However, although the percentage of women who deliver in health facilities increased from 17% to 34% during 1995–2010, facility-based deliveries in Eritrea remain below the SSA average of 48% [18, 19].

We examined the possible effect of Eritrean culture on women’s utilisation of and access to maternal health services (objective 2). We found that Eritrea uses mass media to target negative aspects of traditional cultural beliefs and practices. One of the female decision makers from the Ministry of Health mentioned FGM as one of the negative aspects of traditional cultural practices that the government has been attempting to eradicate. The lead researcher interviewed a decision maker from the Ministry of Health responsible for implementing FGM policies. His task is to conduct regular visits to ANC clinics to remind pregnant women and families about the media campaigns which are conducted by the government which includes a warning that FGM is punishable by law with imprisonment and a 10,000 nakfa (Eritrean currency) fine. Another example that could indicate the effectiveness of mass media in this study is that most of the women who had had a previous pregnancy reutilised maternal health services despite their dissatisfaction with some aspects of the government health services.

Another example of the traditional cultural beliefs that influence the degree to which women access and utilise maternal health services is related to some traditional practices such as belief in the traditional birth attendants (TBAs) [28]. For example, in Ethiopia, it was found that even though 71% of women attended ANC services, 42% believed that seeking facility-based delivery was not necessary, due to influence of TBAs [28]. Although in our study 38 of the 40 women stated that contemporary culture in Eritrea encourages them to access and utilise maternal health services, the country has a relatively low rate of facility based deliveries and home birth is still common [18, 19]. This suggests that belief in traditional cultural practices still exists in Eritrea and more efforts are needed to increase women’s awareness about the significance of childbirth in a health facility.

#### Improvement in gender equality (role of Eritrean women during the two wars with Ethiopia (1961–1991, 1998–2000))

We explored how the two wars in Eritrea (1961–1991, 1998–2000) have influenced women’s status and affected their utilisation of and access to maternal health services (objective 3). We found that Eritrean women’s role as combatants during the War of Independence was mentioned by women as being a major factor in their empowerment, in improving women’s status, and thereby enhancing gender equality. This is significant because empowering women through gender equality has been found to be associated with improvement in maternal health outcomes [43, 44]. In addition, two indicators that could suggest improvement in gender equality were males’ involvement in maternity care and related government regulations that discourage men from divorcing their wives/partners during pregnancy.

Women’s empowerment has been found in countries like Uganda and Burkina Faso to be associated with men’s involvement in their wives/partners attendance at ANC clinics [25]. In our study, all five husbands/partners interviewed indicated that their role is crucial in supporting their wives/partners during pregnancy and childbirth. Government policy that requires men to accompany their wives/partners to ANC clinics during the first trimester for infectious diseases testing as mentioned by some participants could be an indicator of female empowerment.

Women’s political participation and the female labour participation rate are two other indicators that could also explain the improvement in gender equality and women’s empowerment in Eritrea. After independence, Eritrean women gained significant status and formed 50% of the Constitutional Commission during the 1997 ratification process of the constitution [45]. The equal male and female involvement in the ratification process could be due to Eritrean women’s important role as combatants during the War of Independence, during which they made up 33% of the armed forces [12]. Another indicator of women’s empowerment is the labour participation rate [46]. In 2014, the labour participation rate among Eritrean females aged 15 and older was 85% compared to 70% for SSA overall [47].

### Quality of care as a potential barrier to women’s utilisation of and access to parts of maternal health services

Our estimation of quality of care – a difficult issue to assess – is based on the WHO patient-centred approach which focuses on whether or not healthcare providers listen to patients’ concerns as well as the perception of the services provided [27, 48]. For example, we used the lack of ultrasound machines in ANC clinics and the observed shortage of healthcare workers as factors in determining women’s perception of poor quality of care. Shortage of healthcare workers has elsewhere been identified as one of the factors contributing to poor quality of care in Eritrea [17]. Short clinic opening hours, a lack of ultrasound machines and shortage of healthcare workers were the three major reasons why women called for private healthcare independent from that run within the public sector by the government (Table 3). This finding also supports the estimation of Eritrea’s maternal healthcare system as offering a low quality of care.

Although the perception that health care is of poor quality appears to be a barrier to women’s utilisation of and access to maternal health services in Eritrea, a recent study in Eritrea found improvements in quality of care [17]. Women’s reutilisation of government healthcare facilities for consecutive pregnancies also suggests an improvement in quality of care and that poor quality was not a total barrier to women’s utilisation of and access to maternal health services in Eritrea. For example, 11 women who previously delivered in health facilities or had hospital experience related to maternity care reported a positive experience which could be one of the reasons they reutilised the health services.

To our knowledge this study is the first to explore maternal health services in Eritrea while also taking into account the influence of the country’s history of wars. This study has some limitations. First, it is not representative of the whole country since we only interviewed women who lived in Asmara and had access to government health facilities. Second, women who did not utilise maternal health services were not interviewed for this study. Third, there are several factors – such as distance to healthcare facilities and transport – that are likely to have had an effect on women’s utilisation of and access to maternal health services but which are beyond the conceptual framework of this study. Fourth, our interviews with healthcare providers and decision makers did not provide us with information on barriers to maternal health. During those interviews, the comments from most interviewees focused mainly on the facilitators of maternal health services such as improvement in gender equality. This could be due to two reasons: either they have low level of awareness of the barriers mentioned by women or they know about the barriers but chose not to address it as a sensitive issue. Finally, although most of the healthcare providers and decision makers experienced the War of Independence, most of the women did not address the effect of war on utilisation of and access to maternal health services. This is because most of the women were not born or were very young during the War of Independence that ended in 1991. Nevertheless, they acknowledged the improvement in gender equality in the country driven mainly by their mothers and female fighters during the War of Independence.

## Conclusion

This study assessed women and their husbands/partners’ perceptions and the possible effect of contemporary Eritrean culture and the history of war on the utilisation of and access to maternal health services in the country. As well, we examined healthcare providers’ and decision makers’ perspectives. The two key facilitators of women’s utilisation of and access to maternal health services were health education and women’s empowerment driven by their role as combatants during the War of Independence. One main barrier was poor quality of care due to lack of ultrasound machines, short clinic opening hours, and a shortage of healthcare workers. As only a limited number of qualitative studies have been published about maternal health services in war-affected sub-Saharan African countries, our findings especially regarding health education and women’s empowerment could be considered in other war-affected countries similar to Eritrea. Nevertheless, further research is needed to investigate our findings – particularly regarding female empowerment driven by women’s role in combat in relation to their maternal health.

## Abbreviations

ANC: Antenatal care
FGM: Female genital mutilation
MMR: Maternal mortality ratio
SSA: Sub-Saharan Africa
STD: Sexually transmitted disease
